# Comprehensive Die Shear Test of Silicon Packages Bonded by Thermocompression of Al Layers with Thin Sn Capping or Insertions

**DOI:** 10.3390/mi9040174

**Published:** 2018-04-11

**Authors:** Shiro Satoh, Hideyuki Fukushi, Masayoshi Esashi, Shuji Tanaka

**Affiliations:** 1 Micro System Integration Center, Tohoku University, 6-6-01, Aoba, Aramaki, Aoba-ku, Sendai 980-3579, Japan; fukushi@mems.mech.tohoku.ac.jp (H.F.); esashi@mems.mech.tohoku.ac.jp (M.E.); tanaka@mems.mech.tohoku.ac.jp (S.T.); 2 Graduate School of Engineering, Tohoku University, 6-6-01, Aoba, Aramaki, Aoba-ku, Sendai 980-3579, Japan

**Keywords:** thermocompression bonding, vacuum seal, Al–Al bonding, low temperature bonding, shear fracture strength, fracture mechanism, Sn

## Abstract

Thermocompression bonding for wafer-level hermetic packaging was demonstrated at the lowest temperature of 370 to 390 °C ever reported using Al films with thin Sn capping or insertions as bonding layer. For shrinking the chip size of MEMS (micro electro mechanical systems), a smaller size of wafer-level packaging and MEMS–ASIC (application specific integrated circuit) integration are of great importance. Metal-based bonding under the temperature of CMOS (complementary metal-oxide-semiconductor) backend process is a key technology, and Al is one of the best candidates for bonding metal in terms of CMOS compatibility. In this study, after the thermocompression bonding of two substrates, the shear fracture strength of dies was measured by a bonding tester, and the shear-fractured surfaces were observed by SEM (scanning electron microscope), EDX (energy dispersive X-ray spectrometry), and a surface profiler to clarify where the shear fracture took place. We confirmed two kinds of fracture mode. One mode is Si bulk fracture mode, where the die shear strength is 41.6 to 209 MPa, proportionally depending on the area of Si fracture. The other mode is bonding interface fracture mode, where the die shear strength is 32.8 to 97.4 MPa. Regardless of the fracture modes, the minimum die shear strength is practical for wafer-level MEMS packaging.

## 1. Introduction

The chip size of MEMS (micro electro mechanical systems) is continuously shrinking. Such a dramatic size reduction has been achieved by advanced wafer-level packaging and MEMS–ASIC (application specific integrated circuit) integration, where metal-based bonding is a key technology, because it can employ much smaller sealing width than glass frit bonding [1]. In addition, hermetic sealing is possible at allowable temperature of CMOS (complementary metal-oxide-semiconductor) backend process (<400 °C) by metal-based bonding. Au–Au and Au–Sn eutectic are typical metal systems for low temperature (<400 °C) metallic bonding. However, Au is the most serious metal contaminant, and is not allowed to be used in CMOS process lines, and also, Au is expensive. Al is common metal in CMOS process, and not expensive. Al–Ge eutectic has been used for MEMS–CMOS integration [2], but the eutectic temperature of 424 °C is higher than that of CMOS backend process. Al–Al wafer bonding has been reported, and the shear fracture force was 11.7 N (1190 g) for about 1 × 1 mm^2^ chip with a 30 μm wide seal frame, i.e., about 100 MPa in shear stress [3,4]. A shear fracture strength of 30 to 50 MPa by pull testing has been also reported [5]. However, bonding was achieved over 450 °C because of difficulties of plastic deformation and dissolution of surface oxide of Al at the lower temperature. Therefore, low temperature (<400 °C) Al–Al hermetic wafer bonding is still a challenge in this field.

We reported hermetic Al–Al bonding at 330 to 390 °C using Al capped with thin Sn as a bonding metal layer [6,7,8]. Although the metal system contains Sn, the bonding sustainability at a re-flow temperature of Pb-free solder (260 °C) was confirmed. The bonding was achieved by the direct contact of Al at the bonding interface, since Sn becomes liquid with a small amount of Al, and diffuses into Al grain boundaries over the eutectic temperature of 228 °C. In addition, Al does not make any solid solutions and intermetallic compounds at room temperature. We observed very thin bonding interface metal in hermetic samples after bonding. The original thickness of Al was 4.0 to 4.4 μm, but it became only 0.5 to 0.6 μm after bonding, because the Al layer was pressed and squeezed.

As mentioned above, Al–Al bonding was demonstrated below 400 °C, with help from the thin Sn cap layer. However, the bonding strength, which is important for reliability, has not been reported yet. In this study, the shear fracture strength of dies bonded below 390 °C was evaluated by a bonding tester, and the relationship between the die shear fracture strength and fracture mechanism was discussed. As a result, we concluded that Al with thin Sn capping is a practical bonding metal system for the wafer-level hermetic packaging of microsystems.

## 2. Materials and Methods

**Figure 1:**
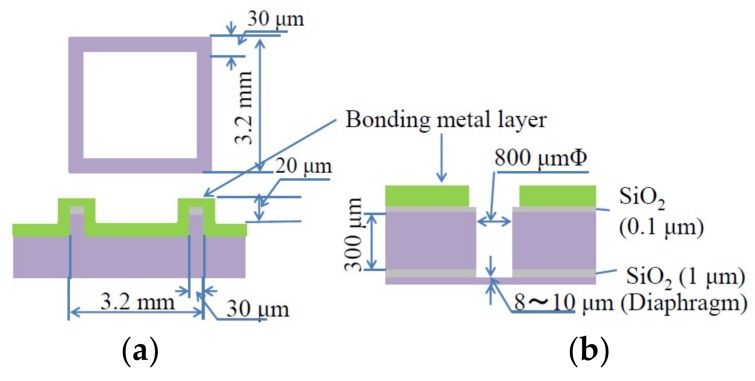

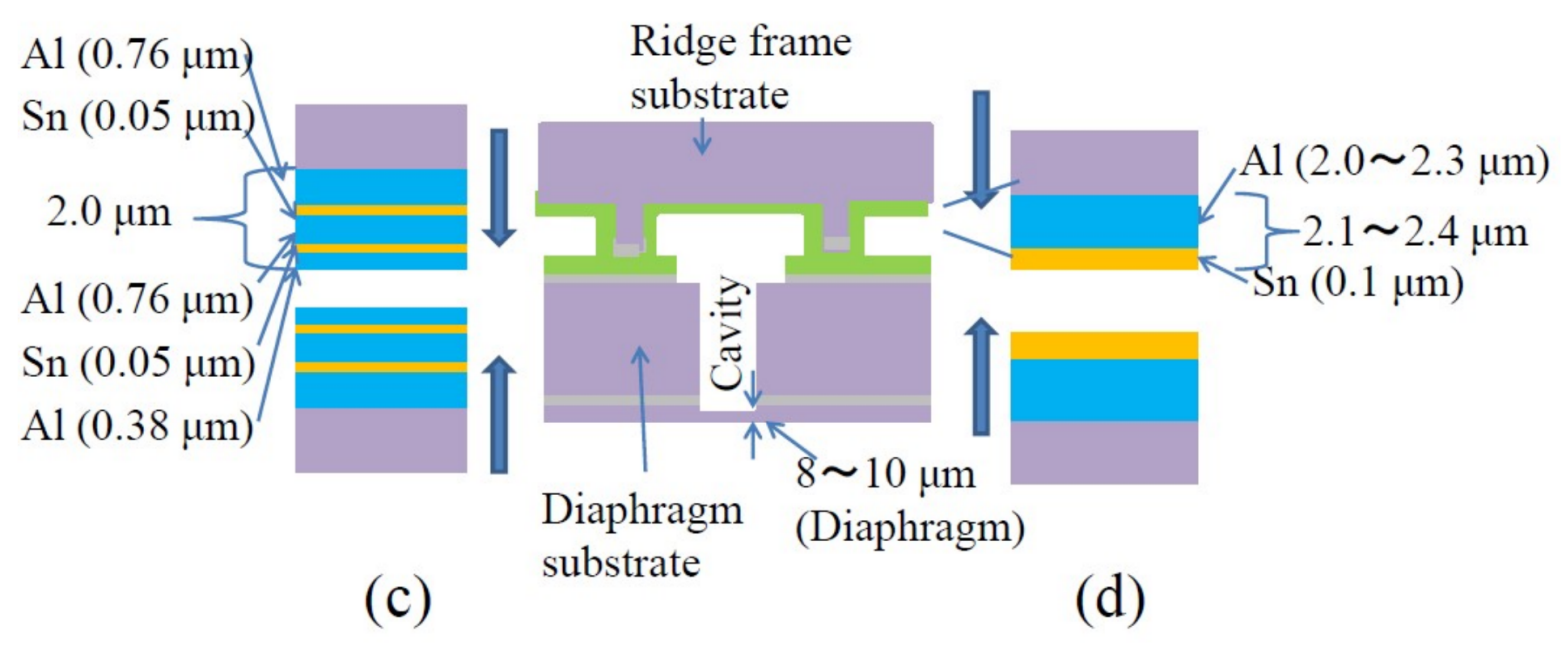
Cross-sectional structure of sample. (**a**) Ridge frame substrate; (**b**) Diaphragm substrate; (**c**) Al bonding layer inserted with thin Sn layers expressed as Al/Sn/Al/Sn/Al//Al/Sn/Al/Sn/Al; (**d**) Al bonding layer capped with thin Sn layer as Al/Sn//Sn/Al.

Figure 1 shows the sample structure. A 2 × 2 cm^2^ Si substrate has 16 sealing ridge frames of 3.2 mm square with 20 μm height and 30 μm width, which is sufficiently narrow compared with that of glass frit bonding (100 to 190 μm in [1], but typically a few hundred μm). The other Si substrate has 16 diaphragms of 800 μm diameter and 8 μm thickness to confirm the hermetic sealing of cavities by their deformation. A pair of substrates are bonded to each other under various conditions. The flat Si substrate, instead of the diaphragm substrate, is also bonded with the ridge frame substrate as die shear fracture strength test sample. Two kinds of bonding layer are used. One is a 1.9 to 2.1 μm thick Al layer with 0.05 μm thick Sn layers inserted, as shown in Figure 1c. The other is a 2 to 2.2 μm thick Al layer capped with 0.1 μm thick Sn as shown in Figure 1d. All the metal layers are continuously sputter-deposited.

**Figure 2:**
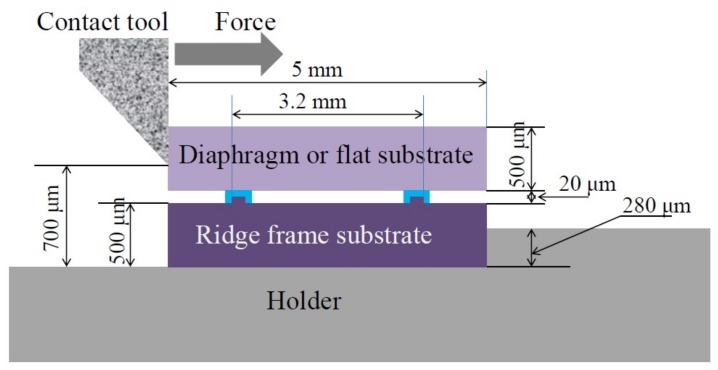
Schematic of bonding tester setup for die shear fracture strength measurement.

These substrates are bonded in vacuum at 360 to 390 °C with a bonding pressure of 43 to 65 MPa using a wafer bonder (SB6e; Süss MicroTec SE; Garching, Germany). Vacuum sealing is checked by the deformation of the diaphragms measured by white light interferometry. After bonding, a 2 × 2 cm^2^ Si substrate is cut into 16 dies by a dicer, and then their shear fracture strengths are measured by a bonding tester (PTR-1101; Rhesca Co., Ltd; Tokyo, Japan). Figure 2 shows the set-up of die shear fracture strength test. The surfaces of a separated die are observed by SEM (scanning electron microscope; Hitachi; Tokyo, Japan), EDX (energy dispersive X-ray spectrometry; Oxford Instruments; Tokyo, Japan) and a surface profiler (Tenkor; Milpitas, CA, USA). In addition, we used FEM (finite element method) analysis to simulate the stress distribution around the ridge structure by die shear test.

## 3. Results

### 3.1. Shear Fracture Strength

**Figure 3:**
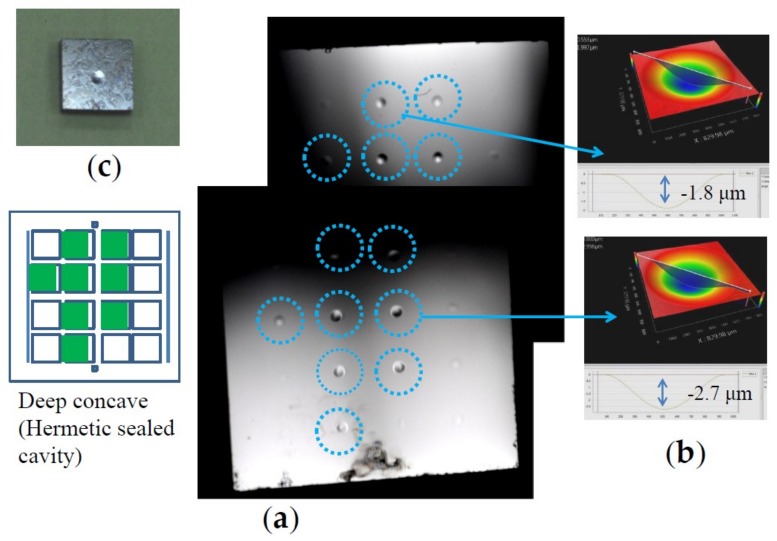
An example of bonded substrate pairs. (**a**) Surface photograph at 22 h after bonding and distribution of sealed dies in the substrate; (**b**) Typical diaphragm deformation of sealed die measured by white light interferometer; (**c**) Sealed die after dicing.

Figure 3 shows a bonded substrate pair, which has the Al bonding layer of Al with thin Sn layer insertion (Figure 1c). The bonding was performed at 360 °C under 60.1 MPa pressure for 2 h. As found in Figure 3a, 8 out of 16 dies were hermetically sealed, since the concave shape of the diaphragm shown in Figure 3b is the result of hermetic sealing [9]. Yield of hermetic seal depends on bonding temperature, pressure, and thickness of bonding metal layer, and in addition, depending on layer structure [6,7,8]. The distribution of non-hermetic seal die in the 2 × 2 cm^2^ Si substrate might be caused by particle contaminations or ununiform bonding pressure. Figure 3c shows the die after saw dicing. The dies with and without the diaphragm were subjected to shear fracture strength measurement.

**Figure 4:**
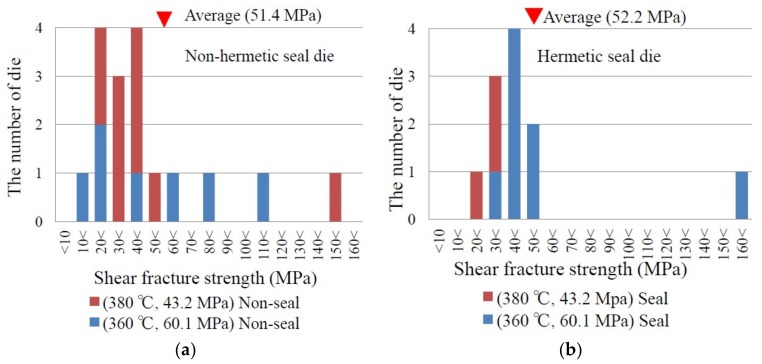
Distribution of shear fracture strength for (**a**) non-hermetic dies and (**b**) hermetic dies. The bonding temperature and pressure, as well as the average of shear fracture strength are shown in each graph.

Figure 4 shows the shear fracture strength distribution of the dies with the bonding layer in Figure 1c (Al/Sn/Al/Sn/Al//Al/Sn/Al/Sn/Al). The substrates were bonded at 360 °C or 380 °C, and the hermeticity of the cavity was checked by the diaphragm deformation. The vertical axis represents the number of dies, and the horizontal axis represents measured shear fracture strength. One group of the dies were not hermetically sealed (Figure 4a), and the other group were hermetically sealed (Figure 4b). Both groups show a similar distribution. The average and standard deviation are 51.4 MPa and 36.2 MPa for the non-hermetic dies, and 52.2 MPa and 37.8 MPa for the hermetic dies, respectively. The minimum shear fracture strength is 19.3 MPa (10.5 N in shear force), which was measured for a non-hermetic die, but that of the hermetic dies is 25.2 MPa.

**Figure 5:**
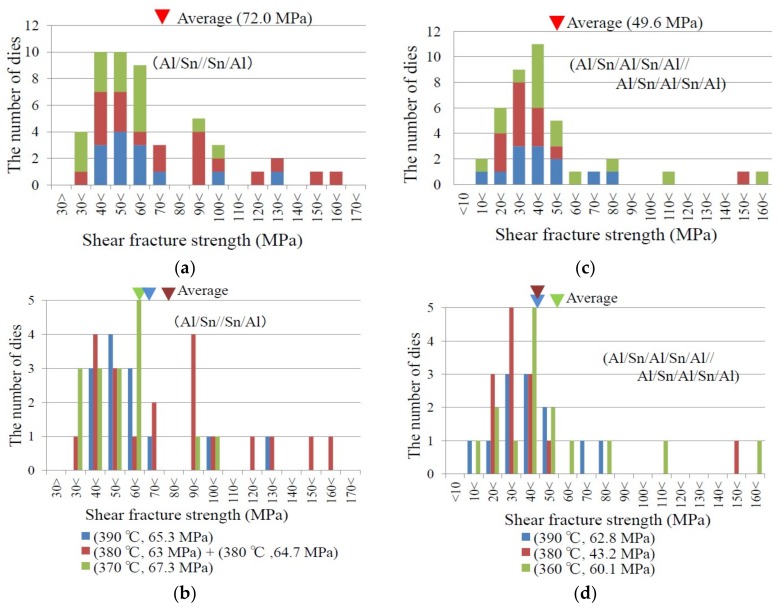
Shear fracture strength distributions for (**a**,**b**) Al/Sn//Sn/Al bonding layer structure and (**c**,**d**) Al/Sn/Al/Sn/Al//Al/Sn/Al/Sn/Al bonding layer structure. The upper graphs (**a**,**c**) show the total number of samples at each strength range regardless of bonding temperature. The lower graphs (**b**,**d**) show the number of samples for each bonding temperature at each strength range. The bonding temperature and pressure are shown in the graphs (**b**,**d**).

Figure 5a,b show the distributions of shear fracture strength of the dies with the bonding layer in Figure 1d (Al/Sn//Sn/Al) bonded at 370 to 390 °C. There was no diaphragm on the dies, and thus, the hermeticity of the cavity did not be confirmed. The average of all dies is 72.0 MPa, and the standard deviation is 31.3 MPa. The average for each bonding temperature is shown in Figure 5b. No clear bonding temperature dependency of shear fracture strength is observed. The minimum shear fracture strength is 32.8 MPa, which was observed for the die bonded at 370 °C.

Figure 5c,d show the distributions of shear fracture strength of the die with the bonding layer in Figure 1c (Al/Sn/Al/Sn/Al//Al/Sn/Al/Sn/Al) bonded at 360 to 390 °C. The average of all dies is 49.6 MPa, and the standard deviation is 32.5 MPa. The minimum shear fracture strength is 19.3 MPa. No clear bonding temperature dependency of shear fracture strength is observed also in Figure 5d. If two bonding layer structures are compared, the Al/Sn//Sn/Al structure exhibits higher shear fracture strength in both average and minimum.

The reason why structure of “Al/Sn//Sn/Al” structure is stronger than that of “Al/Sn/Al/Sn/Al//Al/Sn/Al/Sn/Al” could be due to the difference of the amount of direct contact area of Al between both substrates. The former has wider non-oxidized Al surface in pre-compression. Whereas, the latter is oxidized in pre-compression, and then oxide is partly broken by compression, as observed by TEM (transmission electron microscope; JEOL Ltd; Akishima, Japan) [7]. Therefore, Al direct contact area of bonding interfaces of the former could be wider than that of the latter, and then Al diffusion between bonding interfaces of the former could be more than that of the latter.

### 3.2. Observation Results of Shear-Fractured Surface

**Figure 6:**
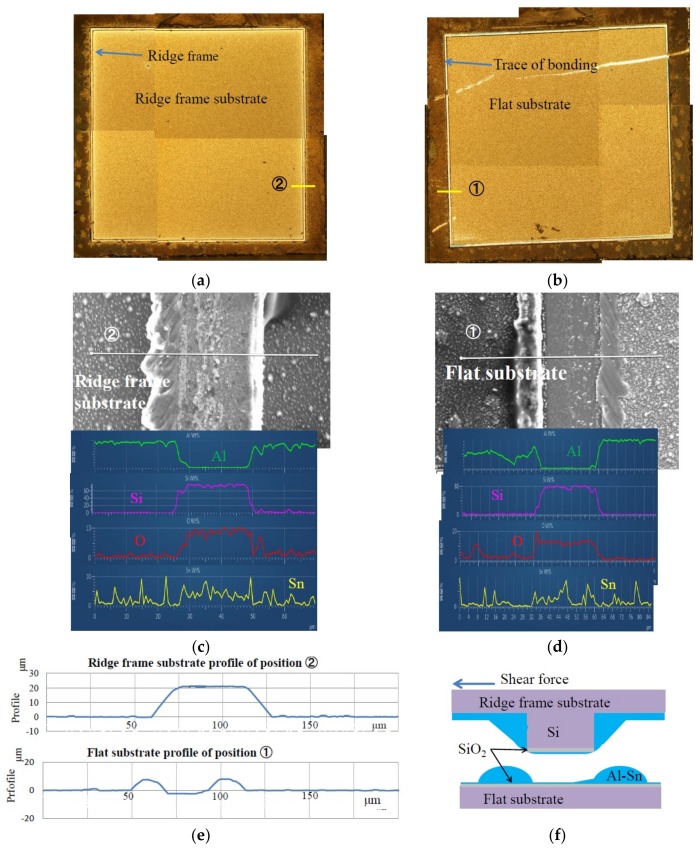
Results of EDX, SEM, and surface profile measurements of shear-fractured dies. (**a**) Surface photograph of ridge frame substrate; (**b**) Surface photograph of flat substrate; (**c**) SEM and EDX results of ridge frame substrate; (**d**) SEM and EDX results of flat substrate; (**e**) Surface profiles of separated substrates; (**f**) Schematic cross section of bonded area after fractured by shear force.

To clarify the relationship between die shear fracture strength and fracture mechanism, we observed the surface of shear-fractured dies by an optical microscope, SEM, EDX, and a surface profiler. Figure 6 shows the observation results of the bonded ridge frame substrate and the opposed flat substrate with the Sn-capped Al bonding layer. The bonding was carried out at 390 °C under 65.3 MPa pressure. The measured die shear fracture strength is 57.1 MPa, which corresponds to a shear fracture force of 20.0 N. Yellow lines in Figure 6a,b represent the observation areas by SEM, EDX, and the surface profiler. In the optical micrographs, the ridge frame and the bonding trace show white color. The color and the EDX analysis suggest that the fractured surfaces mainly consist of silicon oxide. We have already reported the results of SEM and EDX observation of bonding interface cross section after slightly separated by tension stress for bonded die with Al bonding layer capped with thin Sn at 390°C under 77.7 MPa pressure for 2h in Figure 6 of reference [6]. It was observed that the Al bonding interlayer became as thin as a thickness of 0.5 to 0.6 μm and its thin interlayer consisted with Al. According to these results, Al with Sn is supposed to exist on the surface as shown in Figure 6f, although EDX signal from Al was not clearly observed. Figure 6e is the surface topologies of a separated pair of the bonded substrate. The bumps at both sides of the bonded and fractured region is the metal which was squeezed out from the bonding layer [6,7].

**Figure 7:**
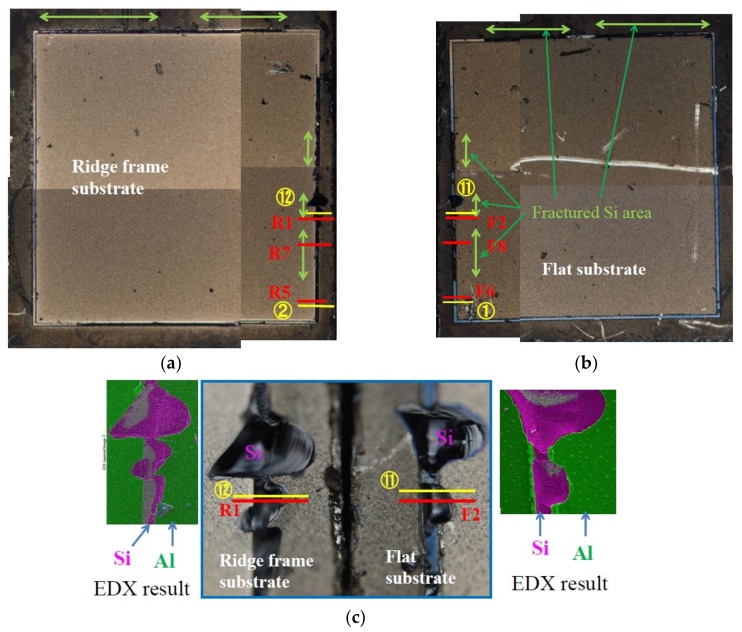

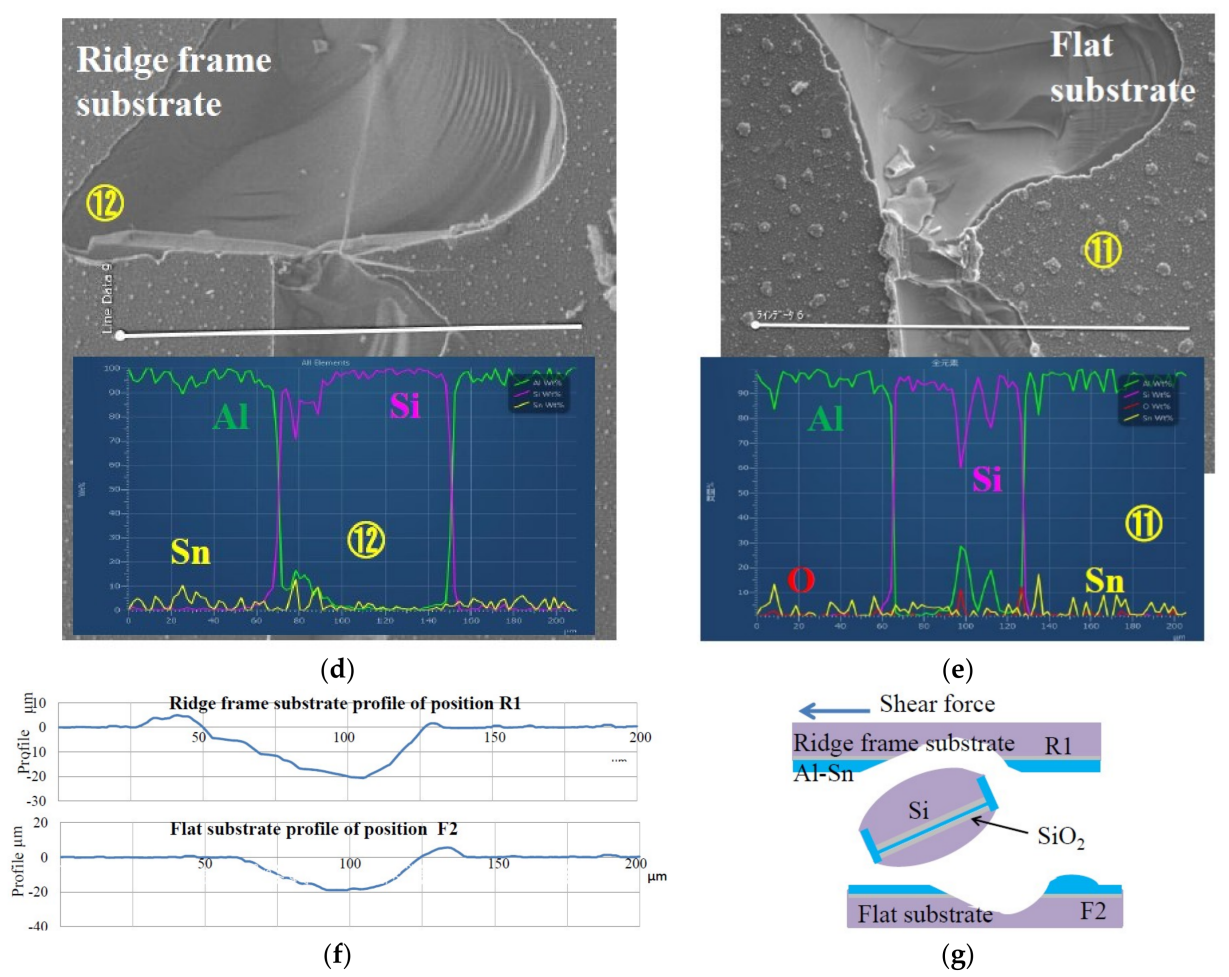
Results of EDX, SEM, and surface profile measurements of shear-fractured dies which showed shear fracture strength of 130 MPa. (**a**) Surface photograph of ridge frame substrate. (**b**) Surface photograph of flat substrate; (**c**) Surface photograph and EDX results of central right part of (**a**) and central left part of (**b**); (**d**) SEM and EDX results of ridge frame substrate; and (**e**) SEM and EDX results of flat substrate; (**f**) Surface profiles of shear-fractured positions R1 and F2; (**g**) Schematic cross section of shear-fractured positions R1 and F2.

Figure 7 shows the surface observation results of the shear-fractured dies, which showed a large fracture strength of 130 MPa (45.6 N in shear fracture force), more than twice larger than that of the above sample. The bonding layer is made of Al capped with thin Sn, and the bonding was carried out at 390 °C under 65.3 MPa pressure for 2 h. Yellow lines and red lines in Figure 7a–c represent the observation areas by SEM and EDX, and the measured position by the surface profiler, respectively. In these figures, there are green arrows and “Fractured Si area”, which will be mentioned later. Figure 7c is the magnified photographs at the center right area of Figure 7a, and the center left area of Figure 7b. The black color areas of the bonding region have concave profile, and mainly consist of Si and a small amount of Al and Sn according to EDX observation (Figure 7d,e). We have already reported that the black color area of a bonding region after forcefully separated by tension stress consisted of fractured Si and that Si of the ridge was tore off to the opposed substrate [6]. On the other hand, the cross-section profiles in Figure 7f show that the bonding regions of both substrates have concave profiles. This is probably because Si around the bonding region of the flat substrate is simultaneously torn off, as schematically depicted in Figure 7g.

**Figure 8:**
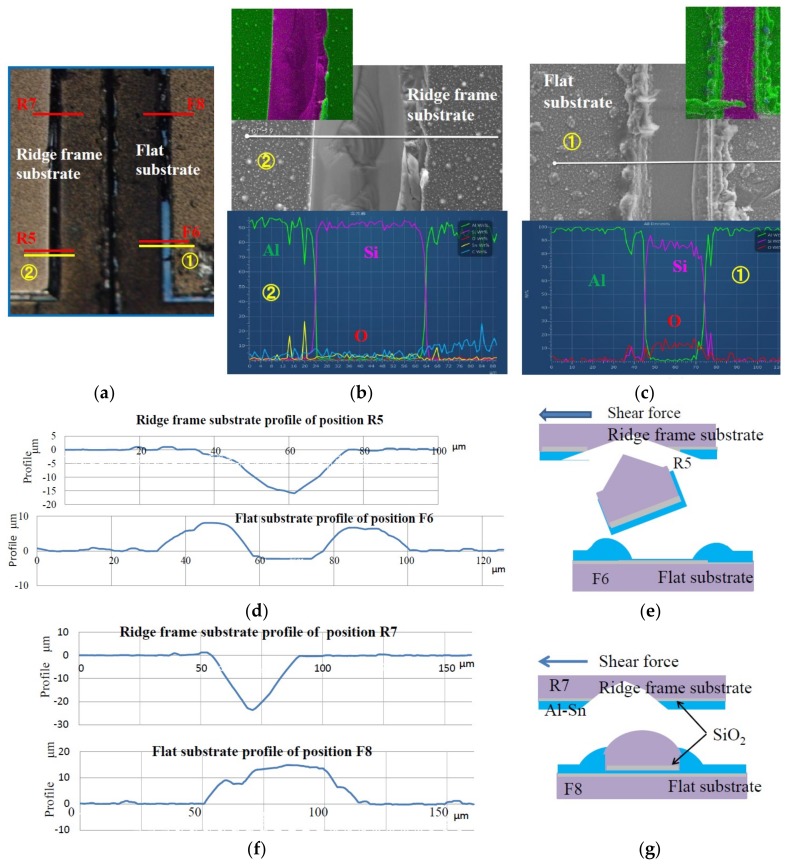
Results of EDX, SEM, and surface profile measurements of shear-fractured die shown in Figure 7. (**a**) Surface photograph of bottom right part of Figure 7a and left part of Figure 7b. (**b**) SEM and EDX observation results of ridge frame substrate and (**c**) of flat substrate. (**d**) Surface profiles of positions R5 and F6; (**e**) Schematically depicted shear fracture cross section around position R5 and F6; (**f)** Surface profiles of positions R7 and F8; (**g)** Schematic cross section of shear-fractured positions R7 and F8.

Figure 8 shows the results of EDX, SEM, and surface profiles of the lower right part of Figure 7a and the lower left part of Figure 7b. The fractured region of the ridge frame substrate at R5 consists of Si, showing black color and concave profile. The EDX signals from O and Al are very small. On the other hand, the fractured region of the flat substrate at F6 shows light blue color and flat profile, accompanying with small bumps on both sides. Furthermore, its surface consists of Si and O judging from EDX analysis, which reveals that the fractured surface is SiO_2_. Since the color of deposited SiO_2_ of 100 nm thickness on the Si substrates was dark blue, light blue color can be explained as the color of SiO_2_ covered with a thin layer like Al, which does not conflict with the previously reported results [6]. Figure 8e shows a predicted fracture mode. Shear fracture which happened in the Al bonding metal and the fracture of the root of the ridge also happened due to the strong impact of shear force.

Figure 8f shows the surface profiles at R7 and F8. The color of both fractured regions is black, and the surface profile of the ridge frame substrate is concave, and that of the flat substrate is largely protruded. These results suggest that shear fracture in Si appeared at the root of ridge, and the fractured Si around the ridge moved to the flat substrate, which agrees with the previously reported results [6,7].

According to the results above mentioned, when the colors of the bonding area of the ridge frame substrate and the opposed flat substrate are black, shear fractures happened in both Si substrates. This mode of fracture is observed when the shear fracture strength is larger than about 42 MPa. In Figure 7a,b, this mode is observed in the regions where green arrows indicate.

**Figure 9:**
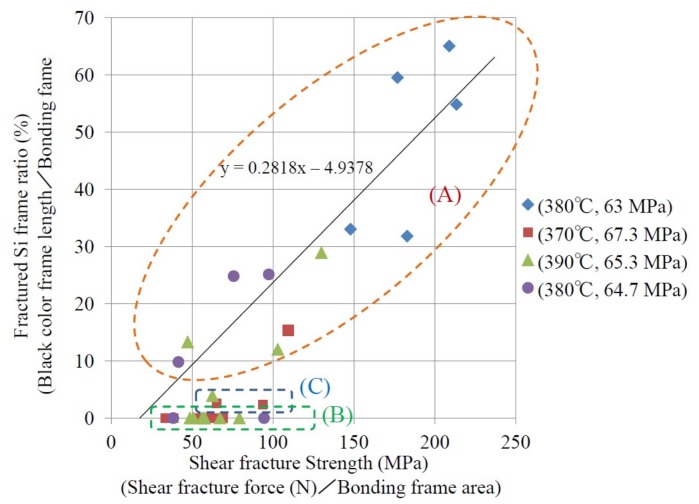
Relationship between fractured Si frame ratio and shear fractured strength. Fractured Si frame ratio (%) is defined as black Si fracture length divided by bonding frame length. The linear approximation formula of region (A), and the bonding temperature and pressure are also represented.

As mentioned above, the Si bulk fracture mode appears when the shear fracture strength is large. Here, the Si fracture length is defined as the length where this fracture mode is observed, i.e., the length of the green arrows in Figure 7a or Figure 7b. Figure 9 shows the die shear fracture strength dependence of the ratio of the Si fracture length to the total length of the ridge frame for the die with bonding metal Al capped by thin Sn layer, which is called fractured Si frame ratio. The obtained results are categorized in three regions. In region (A), the fractured Si frame ratio has a positive linear correlation with the shear fracture strength with an *x*-axis segment of 17.5 MPa. The shear fracture strength ranges from 41.6 to 209 MPa. In regions (B) and (C), the fractured Si frame ratio has no dependence on die shear fracture strength. All the dies in region (B) have white and/or light blue color frames, i.e., frames fractured in the bonding metal. The obtained shear fracture strength ranges from 32.8 to 97.4 MPa. In region (C), the dies have a few % of the fractured Si frame ratio, and the obtained shear strength ranges from 62.6 to 93.8 MPa.

## 4. Discussion

In region (B), fracture happened in the bonding metal, as confirmed by SEM, EDX, and surface profiler observation. In region (C), the fractured Si frame ratio has no dependence on shear fracture strength as shown in Figure 9. Most parts of the frame shows white and/or light blue color, except for a small % of black color parts. Therefore, the fracture mode in region (C) is similar to that in region (B), which is bonding interface fracture mode. The measured shear fracture strength is 32.8 to 97.4 MPa in regions (B) and (C). Itoh et al. reported that the shear fracture strength of diffusion-bonded and annealed 0.5 wt % Sn–Al bulk of 10 × 10 × 5 mm^3^ was around 40 MPa [10], which roughly agree with our experimental results (32.8 to 97.4 MPa).

In region (A), the shear fracture strength is proportional to the fractured Si frame ratio, as shown in Figure 9. In addition, large shear fracture strength over 200 MPa was measured when Si bulk fracture mode was dominant. This shear fracture strength is significantly larger than the shear fracture strength of 0.5 wt % Sn–Al reported in [10]. To explain this result, the following two questions must be answered. (1) Why does the Sn–Al bonding layer stand shear stress over 200 MPa? (2) Is Si fractured by shear stress around 200 MPa or even smaller?

As for the first question, there are two possible reasons. One is that the normal force, as well as shear force, was applied to the interface during the die shear test, and the bonding areas of both sides were strongly pressed and fixed together via the Sn–Al boning layer as thin as 0.5–0.6 μm. Another reason is the work hardening of Al [11] by strong compression.

**Figure 10:**
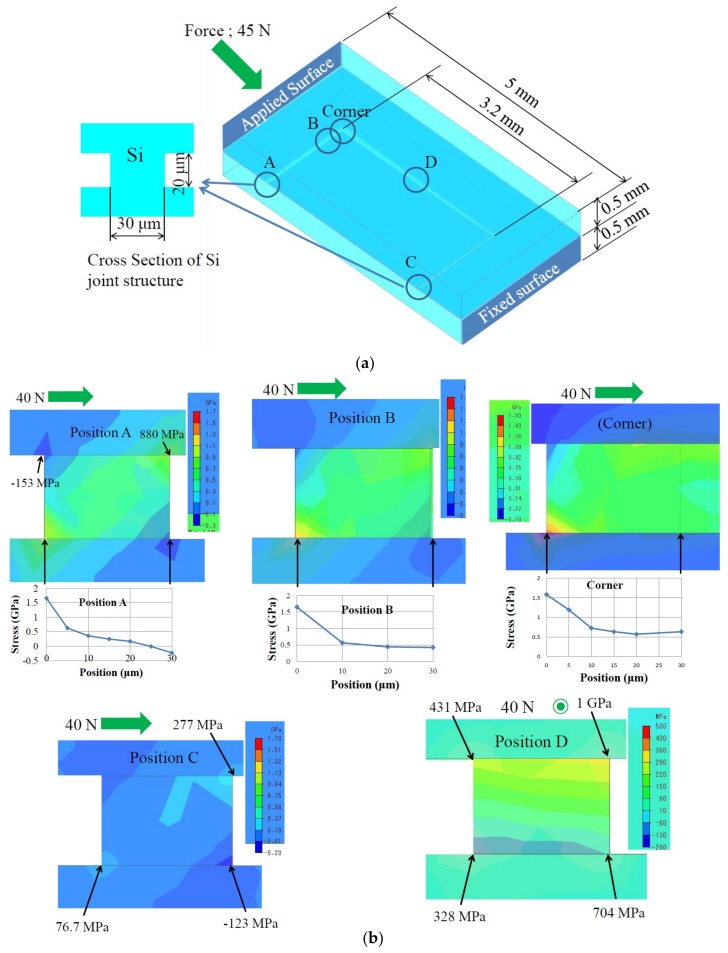
FEM analysis of stress during die shear test. (**a**) Analysis model of sample shown in Figure 1; (**b**) Maximum principal stress in cross sections at positions A to D and “Corner” shown in (**a**).

To consider the second question, FEM (finite element method) analysis was carried out. Figure 10a represents the analysis model, where only a half body is depicted. The bonding metal layer, which is too thin to model using handy mesh sizes, is omitted because the purpose of this analysis is to estimate the stress around the ridge structure. A shear force of 45 N, which is approximately equal to the force applied to the sample shown in Figure 7 and equivalent to a die shear stress of 129 MPa, is applied to the front surface of the upper substrate, and the opposite rear surface of the lower substrate is fixed. This model simulates the setup of the bonding tester shown in Figure 2. The maximum principal stress distributions in the cross sections of the ridge structure are shown in Figure 10b. The observed areas are labeled as A to D and “Corner” as shown in Figure 10a. At the positions A, B, and “Corner”, which are close to the front surface with the force applied, large tensile stress over 1.5 GPa is observed at the outer bottom corner. At the position C near the rear side, stress is relatively small. At the position D near the side, large tensile stress around 1 GPa appears at the outer top corner.

There are many reports on the fracture strength of Si single crystal. The fracture strength in <110> direction has been reported to be from 0.6 to 1.2 GPa, depending mainly on surface defects [12], or even higher around 1.5 GPa [13]. In <100> direction, it has been reported to be 1.73 GPa [14] and 0.59 GPa [15]. Roughly speaking, the fracture strength of Si in micromachined structures is several hundred MPa to 1.5 GPa, depending on surface roughness, process defects etc. Therefore, it is reasonable that the bulk Si fracture mode happens in this experiment, if the Sn–Al bonding layer stands as discussed above. Malik et al. [5] carried out the pull test of Al–Al thermocompression-bonded samples, and found that the bond strength of Al–Al was larger than the strength of crystalline Si. What they found has some similarity to our experimental results.

In this study, the shear fracture strength was widely distributed, and different fracture modes, the bonding interface fracture and bulk Si fracture modes, were observed. The reason for such results is not clear, but the defects of the bonding metal, the Si ridge, and the substrates may have some influence. Some errors in force vectors applied by the die shear tests, i.e., measurement error, may also have an influence. Regardless, the obtained shear fracture strength, 32.8 MPa as the minimum shear fracture stress for the Sn-capped Al layer, is sufficient for practical application.

## 5. Conclusions

In this study, the shear test of Si die pairs bonded by low temperature thermocompression of Al films with thin Sn capping or insertion was carried out. The die shear strength, fracture modes, and their relationship were comprehensively investigated. Two fracture modes, the Si bulk fracture, and the bonding interface fracture mode, were confirmed. The former mode appears due to stress concentration at the corners of a ridge structure, when a very thin Sn–Al bonding layer stands at a relatively higher shear stress, probably because of the interface compression force and the work hardening. The die shear strength of this mode is 41.6 to 209 MPa, proportionally depending on the area of Si fracture. The latter mode is the bonding interface fracture mode, where fracture happens in the bonding layer. The die shear strength of this mode is 32.8 to 97.4 MPa, which roughly agrees with the shear strength of 0.5 wt % Sn–Al bulk. From a practical point of view of wafer-level MEMS packaging [1], the minimum die shear strength is sufficient regardless of the fracture modes.
